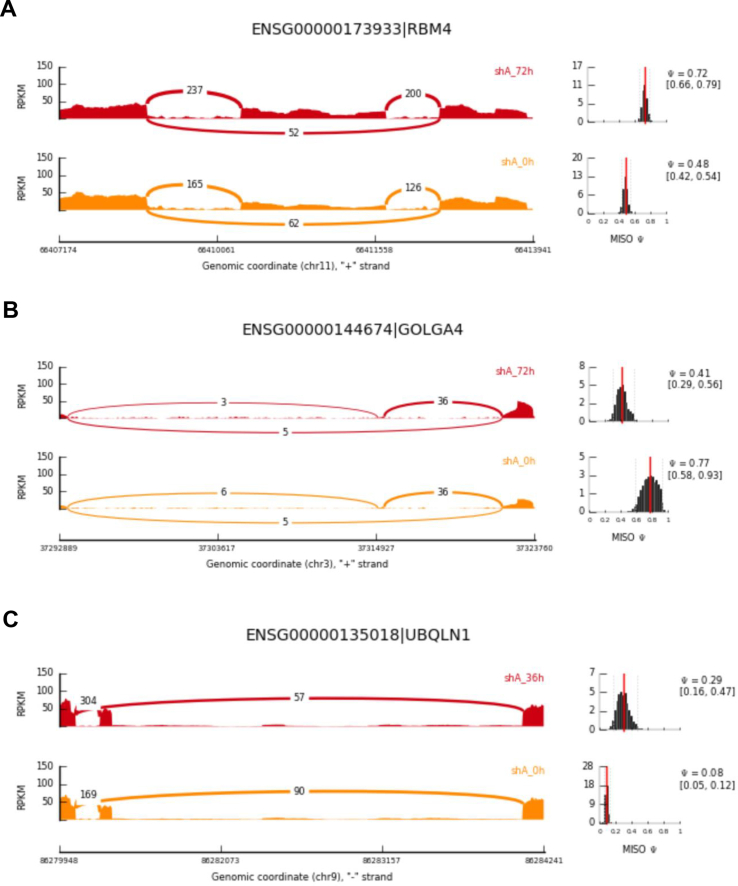# Aurora kinase A regulates cancer-associated RNA aberrant splicing in breast cancer

**DOI:** 10.1016/j.heliyon.2023.e17386

**Published:** 2023-06-24

**Authors:** Sisi Li, Yangfan Qi, Jiachuan Yu, Yuchao Hao, Lingzhi Xu, Xudong Ding, Minghui Zhang, Jingshu Geng

**Affiliations:** a Department of Pathology, Harbin Medical University Cancer Hospital, Harbin, China; b Institute of Cancer Stem Cell, Cancer Center, Dalian Medical University, Dalian, China; c Department of Anesthesiology, The First Affiliated Hospital of Dalian Medical University, Dalian, China; d Department of Oncology, The Second Affiliated Hospital of Dalian Medical University, Dalian, China; e Department of Pathology, The Second Affiliated Hospital of Dalian Medical University, Dalian, China; f Department of Oncology, Chifeng City Hospital, Chifeng, China

**Keywords:** Aurora kinase A, RNA splicing, Splicing factor, Breast cancer

## Abstract

The contribution of oncogenes to tumor-associated RNA splicing and the relevant molecular mechanisms therein require further elaboration. Here, we show that oncogenic Aurora kinase A (AURKA) promotes breast cancer-related RNA aberrant splicing in a context-dependent manner. AURKA regulated pan-breast cancer-associated RNA splicing events including GOLGA4, RBM4 and UBQLN1. Aberrant splicing of GOLGA4 and RBM4 was closely related to breast cancer development. Mechanistically, AURKA interacted with the splicing factor YBX1 and promoted AURKA-YBX1 complex-mediated GOLGA4 exon inclusion. AURKA binding to the splicing factor hnRNPK promoted AURKA-hnRNPK complex-mediated RBM4 exon skipping. Analysis of clinical data identified an association between the AURKA-YBX1/hnRNPK complex and poor prognosis in breast cancer. Blocking AURKA nuclear translocation with small molecule drugs partially reversed the oncogenic splicing of RBM4 and GOLGA4 in breast cancer cells. In summary, oncogenic AURKA executes its function on modulating breast cancer-related RNA splicing, and nuclear AURKA is distinguished as a hopeful target in the case of treating breast cancer.

## 1 Introduction

RNA alternative splicing (AS) exerts an important effect on controlling gene expression and expanding proteomic diversity [[1], [2], [3]]. Aberrant splicing gives rise to abnormal protein isoforms that contribute to tumorigenesis, tumor aggressiveness, and resistance to therapy [[4], [5], [6]]. Deregulation of RNA splicing is implicated in multiple human cancers and modulates cancer cell biology from almost all perspectives concerning multiplication, cell-cycle control, differentiation, metabolism, apoptosis, angiogenesis and metastasis [[7], [8], [9], [10], [11], [12]]. For example, TEAD4-S disrupts the binding of canonical TEAD4-FL to YAP by virtue of the performance of dominant-negative isoform, so as to repress cancer cell proliferation and epithelial-to-mesenchymal transition (EMT) [13]. Abnormal splicing of the ketohexokinase (*KHK*) transcript resulting in the isoform switch from high-activity ketohexokinase-C to low-activity ketohexokinase-A enhances PRPS1-dependent nucleic acid synthesis and inhibits the regulatory role of fructose metabolism regarding the manufacturing of phosphate, ATP, and reactive oxygen species (ROS), thereby driving hepatocellular carcinoma development [14]. Mutations in WT1, the Wilms' tumor suppressor gene, attenuate the expression of the antiangiogenic VEGF(165)b splice isoform and elicit abnormal gonadogenesis, renal failure, and Wilms’ tumors [15]. Compared with mice inoculated with a CD44v(−) subpopulation, mice receiving the CD44v(+) subpopulation of 4T1 breast cancer cells through orthotropic implantation develop lung metastasis with stem-like cancer cell amplification [16]. Nevertheless, the detailed mechanisms underlying the dysregulation of RNA splicing in cancer remain poorly characterized.

AS is mediated under the action of *trans*-acting splicing factors, which bind to *cis*-elements located inside pre-mRNAs and have a strong influence on the selection of splice sites [17]. Dysregulated expression and somatic mutations of splicing factors result in aberrant splicing in cancer [18]. For instance, upregulation of PTB, hnRNPA1 and hnRNPA2 by oncogenic MYC promotes aerobic glycolysis by sustaining a high PKM2/PKM1 ratio in tumor cells [19]. Whole exome sequencing in 105 patients with chronic lymphocytic leukemia identified the mutations in the splicing factor SF3B1, exhibiting relations to faster disease progression and poorer overall survival [20]. Current studies also indicate that transcription factors possess dual functions, that is, combining with RNA sequences neighboring target exons and transactivating splicing regulators controlling such exons at the expression level, thereby directly and indirectly regulating AS [21]. MYC, a well-studied transcription factor, safeguards proper pre-mRNA splicing as an essential step in lymphomagenesis, and disrupting the interaction of MYC with PRMT5 results in either intron retention or exon skipping [22]. The RNA splicing regulatory network is complex, and identifying the key molecules that regulate oncogenic splicing may lead to the design of targeted molecular therapies as a new strategy for cancer treatment.

Aurora kinase A (AURKA) has become a crucial participant in facilitating tumor generation and cancer development [23]. According to prior reports, AURKA in the nucleus performs a function independent of kinase to enhance the phenotype of breast cancer stem cells, where it transactivates target gene expression of MYC and FOXM1 [24,25]. A study conducted almost recently uncovered that nuclear AURKA exerts a pivotal effect on lung cancer tumorigenesis by mediating the interaction between the m^6^A reader YTHDC1 and the splicing factor SRSF3 or hnRNPK to regulate the alternative splicing of RBM4, revealing how a reader of m^6^A modification acts as a switch for an oncogenic splicing event triggered by a tumorigenic signal in lung cancer. However, how oncogenic AURKA poses the impact on modulating breast cancer-related RNA splicing has not been reported. In this study, we show that AURKA promotes breast cancer-related RNA aberrant splicing in a context-dependent manner. AURKA interacted with YBX1 to facilitate GOLGA4 exon inclusion and cooperated with hnRNPK to promote RBM4 exon skipping. The AURKA-splicing factor-aberrant splicing axis presented a relation to breast cancer with adverse outcomes. Blocking AURKA nuclear translocation with small molecule chemicals partially reversed the oncogenic splicing of RBM4 and GOLGA4 in breast cancer cells. Taken together, the discoveries of the present study not only highlight an innovative performance of oncogenic AURKA in controlling pan-breast cancer-associated RNA splicing, but also provide bases for the design of new strategies for breast cancer treatment.

## 2 Materials and methods

### 2.1 Cell culture and chemicals

The American Type Culture Collection (ATCC) supplied HEK293T cell line, breast epithelial cell line MCF-10A as well as human breast cancer cell lines (AU-565, MCF-7, BT-549, SK-BR-3, MDA-MB-231). Prior to procurement from ATCC and after a period of laboratory culture, standard DNA-typing method adopting short tandem repeats was applied to authentication of these cell lines. Based on the suggestion of ATCC, all the cell lines underwent standard medium culture plus 37 °C incubation under 5% CO_2_ through a humidified incubator.

JNJ-165, VX-680, PHA-680632 and MLN8237 (Selleck Chemicals) besides doxycycline (DOX, Clontech) were utilized. Nocodazole and puromycin were purchased from Selleck.

### 2.2 Clinical samples

Patients operated at the Second Affiliated Hospital of Dalian Medical University (Dalian, China) were recruited for acquisition of breast cancer specimens in accordance with informed consent and Institutional Review Board guidelines.

### 2.3 RNA sequencing analysis

Trizol reagent was employed to retrieve total RNA from MDA-MB-231 cells manifesting AURKA downregulation triggered by DOX. Illumina TruSeq Stranded Total RNA with Ribo-Zero Globin kit (Illumina, Inc., San Diego, CA) was utilized for preparation of cDNA libraries. Hi-Seq 2000 platform (Illumina) was used to produce paired-end reads before mapping to human genome. The MISO pipeline was adopted to probe into the variation in splicing isoforms.

### 2.4 Construction besides transfection of plasmids

Inducible pLKO-Tet-On vector was cloned with shRNA fragments specifically aiming at AURKA [26]. Then cloning of human genome cDNA for RBM4 fragments in full length and truncated length as well as pLVX-DsRed-Monomer-N1 vector (Clontech) ligation was implemented, and Flag was labeled. DNA sequencing was performed to examine all vectors for the fidelity. As instructed by the manufacturer, the cell transfection with expression plasmids was executed via lipofectamine 2000 or lipofectamine 3000 (Invitrogen). Supplementary Tab. 3 displayed all primers in the present research.

### 2.5 Lentivirus preparation

Plasmids psPAX2 (Addgene) and pMD2.G (Addgene) in the second-generation packaging lentivirus were chosen for lentivirus packaging in HEK293T cells. The virus titer was determined by ultra-centrifugation of lentivirus concentration and continuous dilution. Finally, Puromycin at the concentration of 2 μg/ml was utilized for screening of cells with lentivirus infected.

### 2.6 RNA interference

GenePharma was responsible for synthesizing and offering RNA interference fragments. Supplementary Tab. 3 listed the target sequences of hnRNPK, YBX1 and control siRNAs.

### 2.7 RT-PCR plus RNA extraction

TRIzol reagent manufactured by Life technologies (No. 15596026) was applied for total RNA acquisition. As per the manufacturer's instructions, cDNA generation was accomplished by means of EasyScript One-Step gDNA Removal and cDNA Synthesis SuperMix Kit purchased from TransGen Biotech (#AE311-03). Besides, reaction system (20 μl) configured with 2 × EasyTaq PCR SuperMix provided by TransGen Biotech (AS111) was introduced to conduct PCR amplification, with internal control determined as ACTB. Supplementary Tab. 3 exhibited the primers for RT-PCR.

### 2.8 CCK8 proliferative testing

Plate (96-well) inoculation plus 37 °C culture under 5% CO_2_ were performed for log-phased cells (2 × 10^3^ cells). Cells in every well were subjected to 4 h of incubation with 10% CCK8 (Selleck) supplemented. A multimode plate reader (PerkinElmer) was applied to examine the 450 nm wavelength for absorbance (OD).

### 2.9 Wound scratch test

The cell migration ability was determined via wound scratch test. MDA-MB-231 cells (5 × 10^5^) underwent 6-well plate seeding as well as marking through 20 μl pipette tips. Monitor abrasions and take photos at designated time points. The space between the two sides of the scratch wound was measured using Image J software.

### 2.10 Tumor growth analysis

MDA-MB-231-vector, RBM4-FL and RBM4-S cells in the same amounts (mixed with PBS at 2 × 10^6^/100 μl) were subcutaneously implanted into 4-6 week-old BALB/C-nu female mice. Next, 4-week tumor formation surveillance was carried out.

Approval was obtained from the Animal Care and Use Committee of Dalian Medical University for all animal studies which were conducted based on authorized protocols in addition to enacted institutional guidelines.

### 2.11 Immunofluorescence analysis

Cells growth for 24 h to a proper confluence was achieved using glass coverslips. Cell permeabilization with 0.5% TritonX-100/PBS together with 1 h of 5% BSA/PBS blocking was performed following 4% (v/v) formaldehyde/PBS fixation. Antibodies were utilized for room-temperature IF. Secondary testing was implemented using anti-IgG antibodies conjugated to Alexa (1:200) provided by Invitrogen Corp. Nuclear staining was carried out on the basis of DAPI (Sigma Aldrich, 1 μg/ml). Image acquisition was realized by virtue of a confocal microscope (Leica).

### 2.12 Western blotting

Protease inhibitors blended with RIPA buffer (prepared using sodium deoxycholate [0.5%], Tris [pH 8.0] at 50 mM, SDS [0.1%], sodium chloride at 150 mM, and NP-40 [1%]) were utilized for sample lysis on ice. Coomassie brilliant blue dye method was adopted to examine the protein concentration. Specifically, each lane with equivalent content of proteins was treated by SDS–PAGE gels (10–15%) before nitrocellulose membrane (Millipore) transfer by means of submerged transfer. Following 1 h of room-temperature sealing with 5% fat-free milk or 3% BSA in TBST, diversified primary antibodies were employed to overnight incubate the membrane at 4 °C. As instructed by the manufacturer, an enhanced chemiluminescence Western blotting kit (K-12045-D50; Apgbio, Beijing, China) was employed for signal visualization subsequent to incubation (1 h, room temperature) with secondary antibodies coupled with peroxidase. The development of blots was detected on the Molecular Imager (Bio-Rad, USA). The following antibodies information was presented: AURKA (Upstate, 07–648), HRP-coupled Goat anti-Rabbit IgG (Thermo-Pierce, 31460), AURKA (Sigma, a1231), P-AURKA (Cell Signaling Technology, D13A11), HRP-labeled Goat anti-Mouse IgG (Thermo-Pierce, 31430), GAPDH (KANGCHEN, KC-5G4), hnRNPK (Cell Signaling Technology, 4675S), YBX1 (Santa Cruz Biotechnology, 398340).

### 2.13 Co-immunoprecipitation analysis

Cultured cells were selected to obtain 500–1000 μg of protein lysates which were overnight incubated at 4 °C with related antibodies (combined with Protein A-G agarose) or simple Glutathione Sepharose beads (GE Healthcare), so as to achieve immuno-complex pull-down. Subsequently, the careful washed samples were supplemented with loading buffer and underwent 10 min of 100 °C boiling. Finally, the proteins subjected to co-immunoprecipitation were inspected by the aforementioned Western blot.

### 2.14 Statistical analysis

Minimally three times of repetition was adopted for each *in vivo* and in *vitro* assay executed in triplicate. Except otherwise specification, means of the three independent assays were utilized to express the data. GraphPad Prism 5.0 (GraphPad Software, Inc.) or SPSS 16.0 software was applied to conduct statistical analyses. ANOVA test plus two-tailed Student's t-test were performed to evaluate the differences in variables. p < 0.05 (*p < 0.05, **p < 0.01, ***p < 0.001) signified differences of statistical significance.

## 3 Results

### 3.1 AURKA modulates pre-mRNA alternative splicing in breast cancer

The role of AURKA in the regulation of RNA splicing was investigated by measuring AS events in mRNAs potentially affected by AURKA by RNA-seq in MDA-MB-231 cells subjected to doxycycline (DOX)-induced AURKA knockdown for 36 and 72 h. 261 AS events in total were observed upon AURKA depletion following an obvious change of percent-spliced-in (PSI) values (PSI ≥0.2), and five types of AURKA-regulated AS events were included as follows: skipped exon (SE), alternative 3′ ss exon (A3E), alternative 5′ ss exon (A5E), mutually exclusive exon (MXE), and retained intron (RI) (Fig. 1A, Supplementary Tab. 1). Subsequent analysis revealed that more than 50% of AS events were negatively regulated by AURKA in SE, A5S, and MXE groups, but not in A3E and RI groups, which suggested that AURKA regulates RNA splicing in a context-dependent manner (Fig. 1B). As revealed by gene ontology analysis on the cellular functions of AURKA-regulated AS events, RNA processing as well as other terms including vesicle-mediated transport, macromolecular assembly, protein localization, cell cycle and regulation of apoptosis exhibited prominent enrichment (Fig. 1C).

**Fig. 1:**
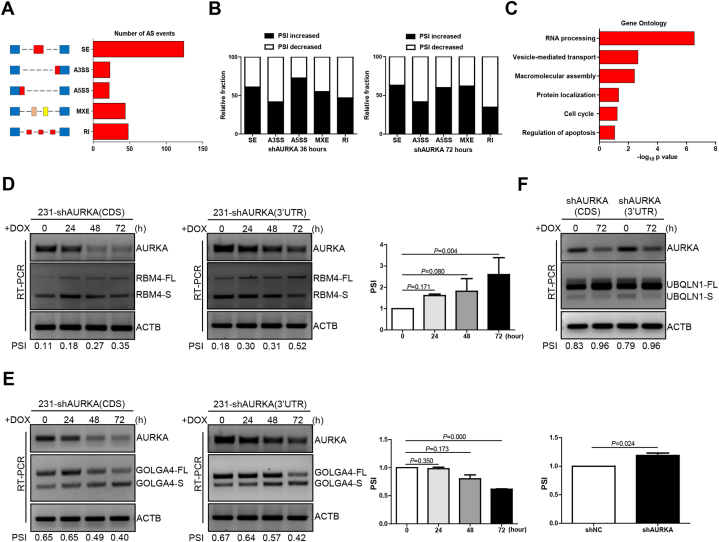
AURKA modulates the RNA splicing switch in breast cancer. **(A)** Quantification of the different alternative splicing (AS) events affected by DOX-induced AURKA knockdown at 36 and 72 h. **(B)** The relative fraction of each AS event positively or negatively affected by AURKA. **(C)** Gene ontology analysis of AURKA-regulated AS targets. **(D**–**F)** Validation of RBM4, GOLGA4, and UBQLN1 splicing switch by RT-PCR in MDA-MB-231 cells subjected to DOX-induced AURKA suppression at different time points. Percent Spliced In (PSI) = FL/FL + S. FL, full-length. S, short. DOX, 2 μg/ml. Data are shown as the mean ± SD. P values were calculated with the two-tailed unpaired Student's t-test and P < 0.05 was considered statistically significant.

Three tumor-associated candidates (RBM4, GOLGA4 and UBQLN1) were selected to validate the RNA-seq data. As a splicing inhibitor, RBM4 regulates tumor-related splicing to perform the functions of tumor suppressor [27]. Alternatively called P230/golgin-245, GOLGA4 takes part in regulatory convey to cell surface from trans-Golgi network (TGN) as a golgin subfamily A member [28]. GOLGA4-RAF1 gene fusion was reported in one case of metastatic melanoma, and correlations of ERK activation and high Ki67 proliferation index with GOLGA4-RAF1 expression were discovered [29]. Ubiquitin 1 (UBQLN1) is a ubiquitin-like protein that transports ubiquitinated proteins to the proteasome and mediates the degradation of ubiquitinated proteins [30]. Loss of UBQLN1 promotes migration and epithelial-to-mesenchymal transition of lung cancer cells [31]. The reads track of RBM4 showed that its PSI value increased from 0.48 to 0.72 at 72 h after DOX-induced AURKA downregulation (Figure S1A). The PSI value of GOLGA4 decreased from 0.77 to 0.41 after 72 h of AURKA knockdown (Figure S1B). The PSI of UBQLN1 increased from 0.08 to 0.29 after 36 h of AURKA depletion (Figure S1C). To confirm the correlation between AURKA and RNA splicing variants of these three candidates, we used DOX-induced Tet-On-shAURKA to silence AURKA in a time-dependent manner in MDA-MB-231 cells. The RT-PCR results showed that the PSI value of both RBM4 and UBQLN1 increased, whereas that of GOLGA4 decreased at 72 h following AURKA depletion in MDA-MB-231 cells (Fig. 1D–F). Treatment with two AURKA kinase inhibitors had no effect on RBM4 splicing in breast cancer cells (Figure S2A). Cell cycle arrest at G2/M phase was associated with the downregulation of RBM4-FL/S variant expression and upregulation of AURKA expression, however, the PSI value of RBM4 did not change compared with that of the control group (Figure S2B). These results indicate that AURKA is a critical player in the regulation of RNA splicing via its kinase-independent activity in breast cancer.

### 3.2 Dysregulation of splicing events involved in breast cancer

We first selected series of breast cancer cells to measure the relative levels of RBM4 and GOLGA4 variants, for the purpose of exploiting the effect of the RBM4 and GOLGA4 splicing switch on tumor biology. It was discovered that in comparison to that in noncancerous breast epithelial cell line (MCF-10A), the RBM4 PSI value was decreased in breast cancer cells (MCF-7, SK-BR-3, AU-565, BT-549, and MDA-MB-231) (Fig. 2A). The PSI value of GOLGA4 was significantly higher in breast cancer cells than in MCF-10A group (Fig. 2A). Next, we measured the relative levels of RBM4 and GOLGA4 variants in clinical breast cancer specimens. Concerning the relative mRNA levels, tumor tissue manifested higher AURKA and RBM4-S but lower RBM4-FL than adjacent normal tissue (Fig. 2B). By contrast, the PSI value of GOLGA4 was higher in tumor tissue than in normal tissue (Fig. 2C). These results indicate that RBM4 tends to be abnormally spliced into the RBM4-S isoform and GOLGA4 tends to be abnormally spliced into the GOLGA4-FL isoform in breast cancer.

**Fig. 2:**
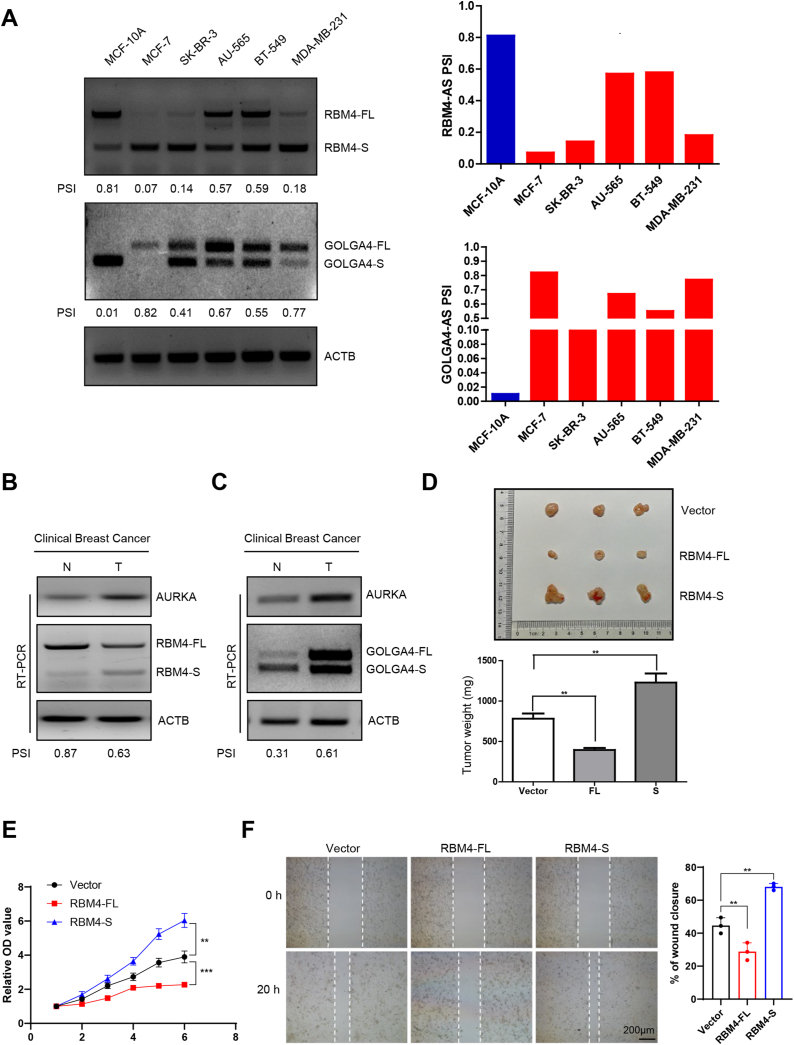
Aberrant splicing of RBM4 and GOLGA4 is closely related to breast cancer development. **(A)** Relative mRNA abundance of RBM4, and GOLGA4 splicing in MCF-10A and other breast cancer cell lines was examined by RT-PCR. **(B–C)** Validation of RBM4 and GOLGA4 splicing switch in clinical breast cancer specimens by RT-PCR. N, normal; T, tumor. **(D)** Immunodeficient mice were subcutaneously inoculated with equal numbers of MDA-MB-231-vector, RBM4-FL, and RBM4-S cells (2 × 10^6^ cells per mouse, n = 3). Photograph of tumors and tumor weights are shown. **(E)** MDA-MB-231 cells were stably transfected with vector, RBM4-FL, RBM4-S plasmids, and the proliferative ability of cells was analyzed by cell counting kit-8 (CCK8) assay. **(F)** The migration ability of cells was analyzed by wound healing assay. Data are shown as the mean ± SD. P values were calculated with the two-tailed unpaired Student's t-test. *P < 0.05, **P < 0.01, ***P < 0.001.

Aiming at verifying the influence on tumor growth exerted by the two isoforms of RBM4, nude mice [BALB/C (nu/nu), female, 4–6 weeks old, n = 3 for every group] were enrolled to receive vector, RBM4-FL and RBM4-S overexpressing MDA-MB-231 cells (2 × 10^6^ cells) through subcutaneous injection in equal number. Following weekly measurement of tumor xenografts for 4 weeks, mice were killed. Tumor weight was significantly lower in mice inoculated with RBM4-FL than in those bearing vector-transfected cells, whereas a dramatically elevated growth rate of RBM4-S cell-derived tumors was observed in contrast with that of from vector cell-derived tumors (Fig. 2D). Based on the results of *in vitro* CCK8 and wound healing assays for breast cancer, cell proliferation and migration was restrained by overexpressed RBM4-FL but facilitated through RBM4-S (Fig. 2E–F). All the findings imply that aberrant RNA splicing of RBM4 and GOLGA4 is closely related to breast cancer progression.

### 3.3 AURKA cooperates with different splicing factors to co-regulate cancer-related RNA splicing

The mechanism underlying the role of AURKA in modulating cancer-associated RNA splicing was examined by overlapping AURKA-interacting proteins published in Biogrid [32] with core spliceosomal proteins [33] (Supplementary Tab. 2). Twenty-three proteins were identified including YBX1 and hnRNPK (Fig. 3A). A co-IP assay confirmed that endogenous AURKA interacted with both YBX1 and hnRNPK (Fig. 3B–C). Correlation analysis in GEPIA indicated that AURKA at a high level has linkage to both YBX1 and hnRNPK with increased expression in breast cancer (Fig. 3D–E). Knockdown of AURKA in MDA-MB-231 cells downregulated the expression of YBX1 (Fig. 3F), whereas it had no effect on the protein expression of hnRNPK (Fig. 3G). RT-PCR was conducted to examine YBX1 and hnRNPK implicated in RNA splicing regulation by virtue of AURKA mediation, which showed that YBX1 knockdown promoted GOLGA4 exon skipping, whereas hnRNPK knockdown promoted RBM4 exon inclusion in breast cancer cells (Fig. 3H–I). However, depletion of YBX1 did not impact RBM4 splicing, and similarly, hnRNPK suppression had no effect on the splicing of GOLGA4 in MDA-MB-231 cells (Figure S3A-B). The aforementioned results hint that AURKA regulates breast cancer-associated RNA splicing by recruiting different splicing factors to form protein complexes.

**Fig. 3:**
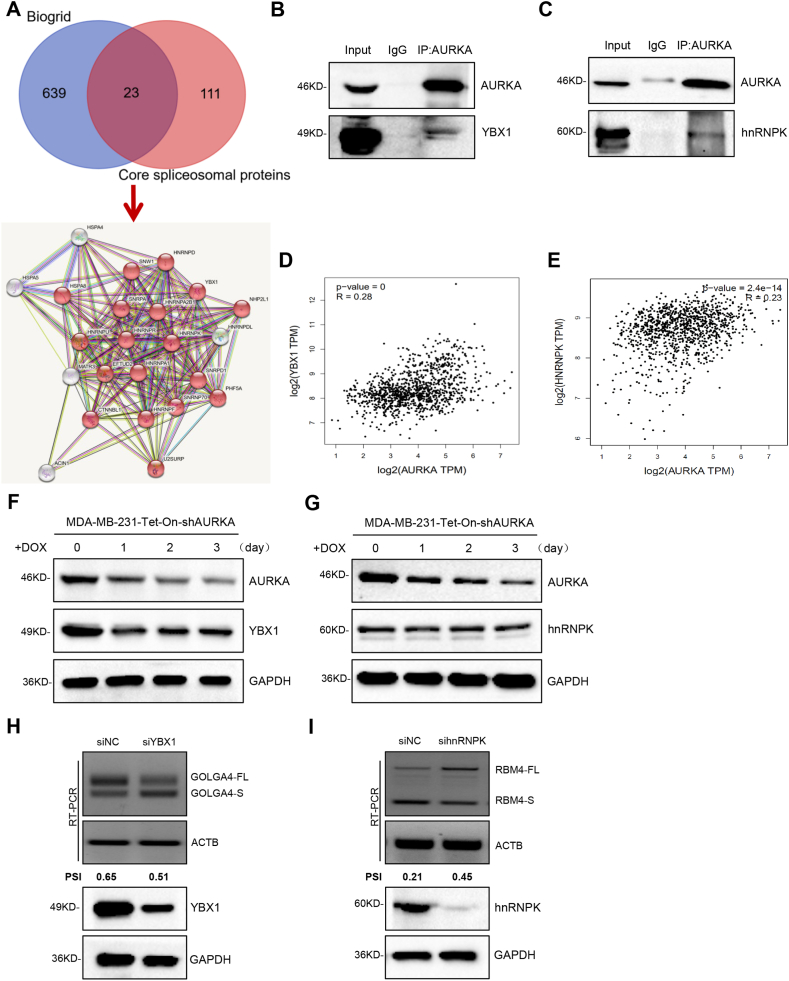
AURKA cooperates with different splicing factors to co-regulate cancer-related RNA splicing. **(A)** Comparison of AURKA interacting proteins from Biogrid with core spliceosomal proteins identified 23 candidates. **(B–C)** The interaction between endogenous AURKA and YBX1/hnRNPK protein was analyzed by co-IP in MDA-MB-231 cells. **(D**–**E)** Analysis of the association between AURKA and YBX1/hnRNPK expression in breast cancer patients from GEPIA. **(F**–**G)** YBX1 and hnRNPK protein expression levels in DOX-induced AURKA knockdown MDA-MB-231 cells were measured by western blotting. **(H)** The GOLGA4 RNA splicing switch was detected by RT-PCR after silencing YBX1 in MDA-MB-231 cells. **(I)** The RBM4 RNA splicing switch was detected by RT-PCR after silencing hnRNPK in MDA-MB-231 cells.

### 3.4 The AURKA-YBX1/hnRNPK complex is associated with poor prognosis in breast cancer

The AURKA-YBX1/hnRNPK complex in breast cancer was examined for its clinical significance by analyzing the correlation between AURKA, YBX1, and hnRNPK protein or mRNA expression levels and relapse-free survival, post progression survival, and overall survival using the survival analysis tools GEPIA and Kaplan-Meier Plotter. AURKA mRNA expression levels were higher in breast cancer tissues than in normal tissues (Fig. 4A). High AURKA mRNA expression was significantly associated with decreased relapse-free survival and post progression survival in breast cancer patients (Fig. 4B). Moreover, patients suffering from breast cancer had decreased OS with YBX1 at high mRNA and protein expression (Fig. 4C–D). Likewise, such patients manifested shortened OS, RFS and PPS significantly related to high hnRNPK expression (Fig. 4E–F). Analysis of AURKA and hnRNPK protein expression levels in clinical breast cancer samples showed that hnRNPK expression was positively correlated with AURKA expression levels (Fig. 4G). Taken together, these data demonstrate that the AURKA-YBX1/hnRNPK complex is associated with adverse outcome in breast cancer.

**Fig. 4:**
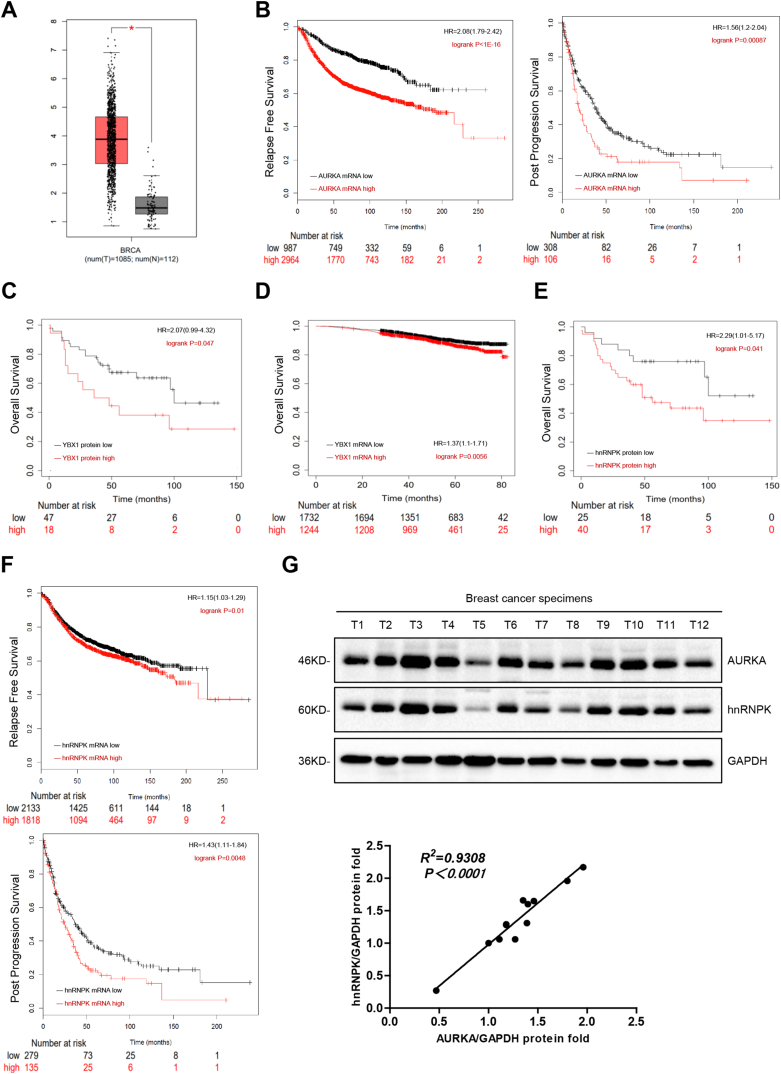
The AURKA-YBX1/hnRNPK complex is associated with poor prognosis in breast cancer. **(A)** AURKA mRNA expression analysis in clinical breast cancer and normal tissues from GEPIA. Tumor: 1085; Normal: 112. **(B)** Correlation analysis of AURKA mRNA expression with relapse free survival (RFS) and post progression survival (PPS) in clinical breast cancer specimens using GEPIA. Total number of patients in the RFS group, 7816; total number of patients in the PPS group, 554. **(C)** Correlation analysis of YBX1 protein expression with overall survival (OS) in clinical breast cancer specimens using Kaplan-Meier Plotter. n = 108. **(D)** Correlation analysis of YBX1 mRNA expression with OS in clinical breast cancer specimens using Kaplan-Meier Plotter. n = 9409. **(E)** Correlation analysis of hnRNPK protein expression with OS in clinical breast cancer specimens using Kaplan-Meier Plotter. n = 108. **(F)** Correlation analysis of hnRNPK mRNA expression with RFS and PPS in clinical breast cancer specimens using GEPIA. Total number of patients in the RFS group, 7816; total number of patients in the PPS group, 554. **(G)** AURKA and hnRNPK protein expression in breast cancer specimens (n = 12; T: tumor) was subjected to Western blot analysis. AURKA and hnRNPK expression levels were normalized to GAPDH and the results of linear regression analysis are shown. R^2^ = 0.9308, P < 0.0001.

### 3.5 Small molecule inhibitors of AURKA nuclear translocation prevent RBM4 aberrant splicing in breast cancer cells

The effect of the small molecule inhibitors JNJ-165 and PHA-680632 on AURKA nuclear translocation in breast cancer cells was examined by immunofluorescence assays, which showed that the two inhibitors promoted the cytoplasmic accumulation of AURKA in MCF-7 cells (Fig. 5A). Then western blotting was utilized to explore how JNJ-165 and PHA-680632 affected AURKA expression. AURKA kinase activity, instead of total AURKA expression, was restrained by PHA-680632 and JNJ-165 according to the data (Fig. 5B). RT-PCR analysis showed that PHA-680632 enhanced RBM4 exon inclusion and GOLGA4 exon skipping in MCF-7 cells, whereas RBM4 and GOLGA4 splicing was not under the impact of JNJ-165 (Fig. 5B). These data suggest that AURKA nuclear translocation inhibitors have the potential to correct RNA aberrant splicing in breast cancer.

**Fig. 5:**
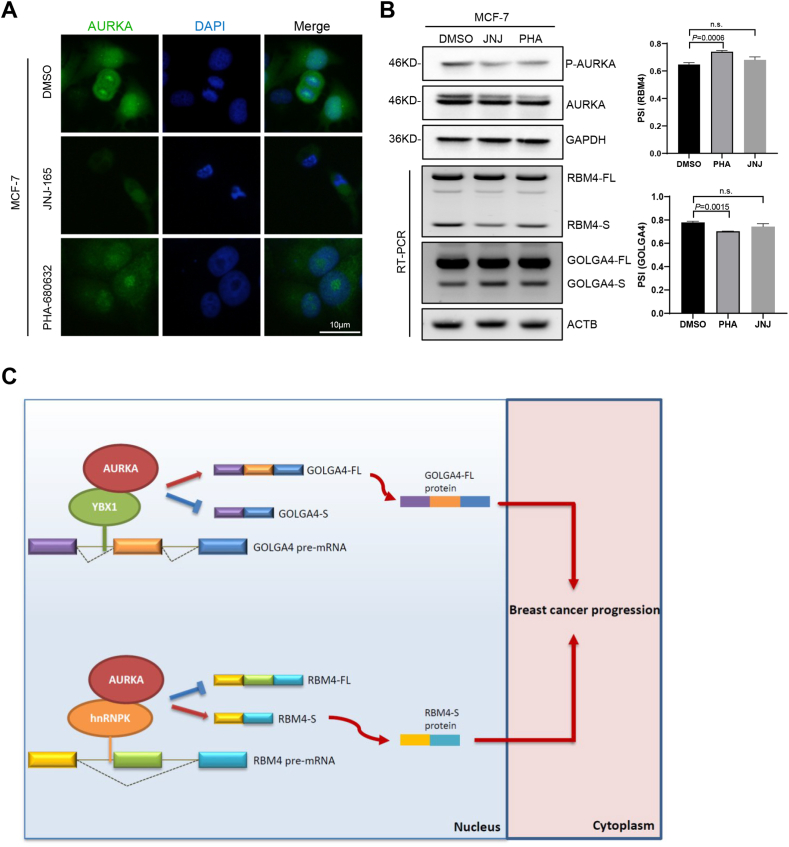
Small molecule inhibitors of AURKA nuclear translocation prevent RBM4 aberrant splicing in breast cancer cells. **(A)** The localization of endogenous AURKA protein in MCF-7 cells treated with DMSO, JNJ-165, and PHA-680632 was determined by immunofluorescence. JNJ-165, 10 μM; PHA-680632, 10 μM. Scale bar, 10 μm. **(B)** P-AURKA and AURKA protein levels in MCF-7 cells treated with DMSO, JNJ-165, and PHA-680632 were measured by western blotting. Validation of RBM4 and GOLGA4 splicing switch by RT-PCR. **(C)** Schematic diagram of the underlying mechanism: AURKA promotes breast cancer-related RNA aberrant splicing in a context-dependent manner. AURKA interacts with the splicing factor YBX1 to promote the generation of the GOLGA4-FL isoform. AURKA cooperates with the splicing factor hnRNPK to mediate the production of the RBM4-S isoform. Data are shown as the mean ± SD. P values were calculated with the two-tailed unpaired Student's t-test and P < 0.05 was considered statistically significant.

## 4 Discussion

In the present study, we show that oncogenic AURKA promotes breast cancer-related RNA aberrant splicing in a context-dependent manner (Fig. 5C). The novel findings of the study were as follows: (a) AURKA modulates pre-mRNA alternative splicing in breast cancer (Fig. 1); (b) Aberrant splicing of RBM4 and GOLGA4 is closely related to breast cancer development (Fig. 2); (c) AURKA cooperates with different splicing factors to co-regulate cancer-related RNA splicing (Fig. 3); (d) The AURKA-YBX1/hnRNPK complex is associated with adverse outcome in breast cancer (Fig. 4); (e) Small molecule inhibitors of AURKA nuclear translocation prevent RBM4 and GOLGA4 aberrant splicing in breast cancer cells (Fig. 5).

Aberrant splicing leads to the production of anomalous proteins resulting in cancer development, although the functional significance of cancer-specific AS events remains largely unexplored. We first discovered and reported that a truncated isoform (RBM4-S), compared to full-length isoform (RBM4-FL), contains only an N-terminal RNA-binding domain [34]. The newly identified RBM4-S isoform promotes lung tumor progression by diminishing RBM4-FL-mediated inhibition of SRSF1-mTORC1 activity [34]. In this study, we found that RBM4 can also be abnormally spliced into RBM4-S isoform in breast cancer cell lines together with clinical breast cancer specimens (Fig. 2A–B). Furthermore, *in vitro* CCK8 and wound healing assays combined with *in vivo* subcutaneous tumor formation assay showed that overexpressing RBM4-FL repressed, whereas RBM4-S enhanced, the proliferation and migration of breast cancer cells (Fig. 2D–F). Moreover, GOLGA4 can be abnormally spliced into GOLGA4-FL isoform in both breast cancer cell lines and clinical breast cancer specimens (Fig. 2A and C), suggesting that aberrant splicing of GOLGA4 has an intimate association with breast cancer progression. Nevertheless, the regulatory effects of the two isoforms of GOLGA4 on tumor initiation and progression have not been clearly confirmed.

Recent work from our group demonstrated that AURKA interacts with hnRNPK in the nucleus and functions as a transcription factor in a complex that induces a shift in MYC promoter usage and activates the MYC promoter [24]. Many transcription factors are linked to splicing control in a direct or indirect regulatory manner [21]. For example, arginine and glutamate rich 1 (Arglu1) is a transcriptional co-regulator that is essential for estrogen receptor-mediated gene transcription and breast cancer cell growth; it also contains an arginine-serine (RS) domain, which suggests that Arglu1 plays a regulatory role in pre-mRNA splicing [35,36]. Knockdown of Zfp871 causes a nearly 40% reduction in Srrm4 mRNA levels, and Zfp871 directly binds to intronic sequences adjacent to its target exons, preferentially promoting neural-enriched exon inclusion in N2A cells [21]. Here we found that AURKA could not regulate the expression of hnRNPK, but could recruit hnRNPK to form splicing co-factors that facilitate RBM4 exon skipping in breast cancer (Fig. 3C, G, 3I). Our recent study demonstrated that hnRNPK facilitates RBM4 exon skipping by combining with the splice site (TCCCTA) of RBM4 pre-mRNA [34]. Since AURKA itself is not an RNA-binding protein, it needs to combine with hnRNPK to form a complex that utilizes hnRNPK's RNA-binding ability to participate in RNA splicing regulation. However, it is unclear whether AURKA itself has RNA splicing regulatory activity. Taken together, these data indicate that a complex formed by oncogenic transcription factors/co-factors may play a direct role in splicing modulation, further expanding the suggestion that targeting the AURKA-hnRNPK axis is a possible therapeutic intervention to improve the prognosis of patients with breast cancer.

Although AURKA kinase inhibitors (e.g., MLN8237, VX680) have shown efficacy in the treatment of cancer [37,38], therapeutic resistance limits their effect [39]. The kinase-independent function of AURKA is considered as a key point of drug resistance and tumor relapse, which is supported by our previous findings that AURKA acquires *trans*-activating activity in a kinase-independent manner to promote breast cancer stemness when translocating into the nucleus, leading to kinase inhibition resistance [24,25]. CD532, an AURKA kinase-independent confirmation-specific inhibitor, disrupts the AURKA-MYCN complex and drives the proteasomal degradation of MYCN in MYCN-driven cancers [40], suggesting a strategy to overcome AURKA kinase-independent oncogenic activity by disrupting protein-protein interactions. Here, we tested an alternative therapeutic approach by blocking the nuclear localization of AURKA. The small molecule chemicals JNJ-165 and PHA-680632 prevent AURKA from translocating into the nucleus [34]. PHA-680632 significantly promoted the inclusion of RBM4 exon in A549, NCI–H460 and MCF-7 cells. However, JNJ-165 had a significant effect only in A549 and NCI–H460 cells, but not in MCF-7 cells (Fig. 5A–B) [34], which may be due to the distinct drug sensitivity of different tissues.

In summary, we demonstrated that oncogenic AURKA promotes breast cancer-related RNA aberrant splicing in a context-dependent manner. The present findings suggest that targeting AURKA nuclear translocation is a promising strategy for overcoming resistance to AURKA kinase inhibitors.

## Data availability statement

Data included in article/supp. material/referenced in article.

## Author contributions

Sisi Li: Conceived and designed the experiments; Analyzed and interpreted the data. Minghui Zhang; Jingshu Geng: Conceived and designed the experiments; Wrote the paper.

Yangfan Qi, Jiachuan Yu,: Performed the experiments; Analyzed and interpreted the data.

Yuchao Hao; Lingzhi Xu; Xudong Ding: Performed the experiments; Contributed reagents, materials, analysis tools or data.

## Funding

This research work was supported by the 10.13039/501100001809National Natural Science Foundation of China (No. 82103659 to S-SL, No. 8210113819 to Y-FQ), Postdoctoral Foundation of Hei Long Jiang Province (No. LBH-Z20074 to S-SL), Harbin Medical University Doctor Green Seedling Ground-breaking Project (No. QMPT-1909 to S-SL). This study was also supported by the 10.13039/501100004763Natural Science Foundation of Inner Mongolia (No. 2020MS08084 to MZ) and the Inner Mongolia Science & Technology Plan Project (No. 2020GG0297 to MZ).

## Declaration of competing interest

The authors declare that they have no known competing financial interests or personal relationships that could have appeared to influence the work reported in this paper.

## Figures and Tables

**figs1:**
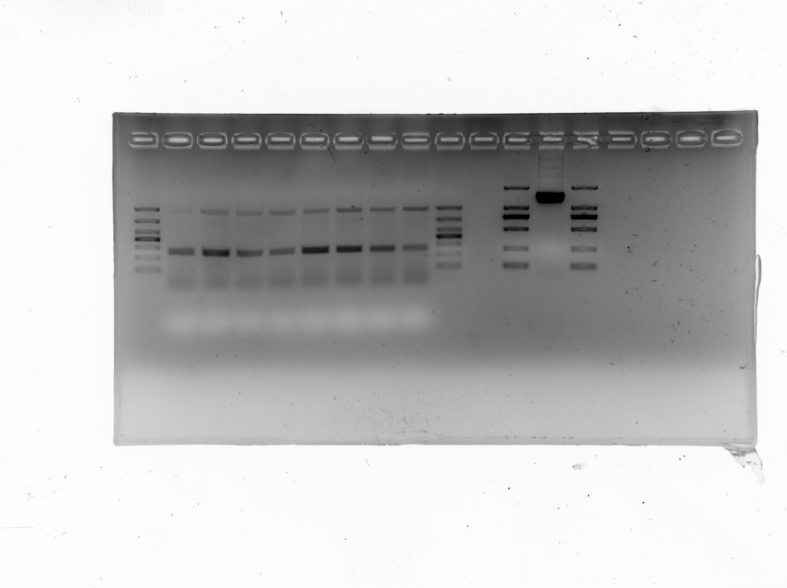


**figs2:**
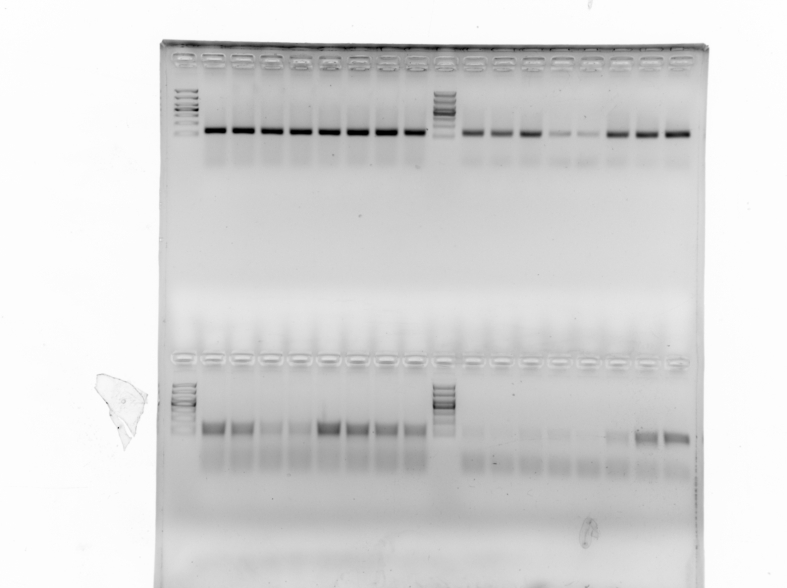


**figs3:**
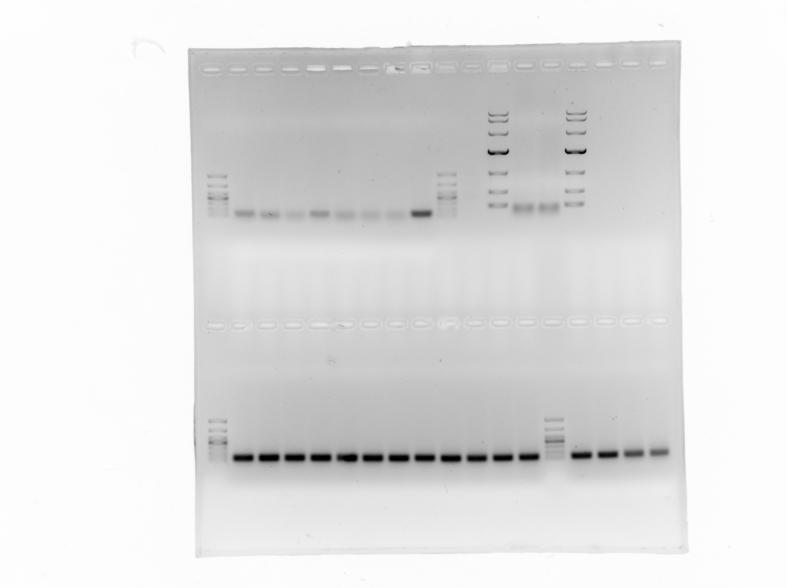


**figs4:**
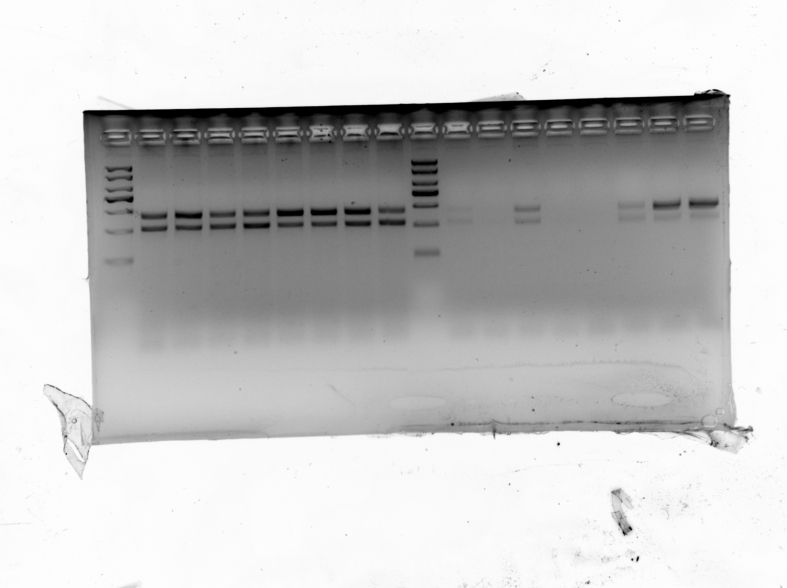


**figs5:**
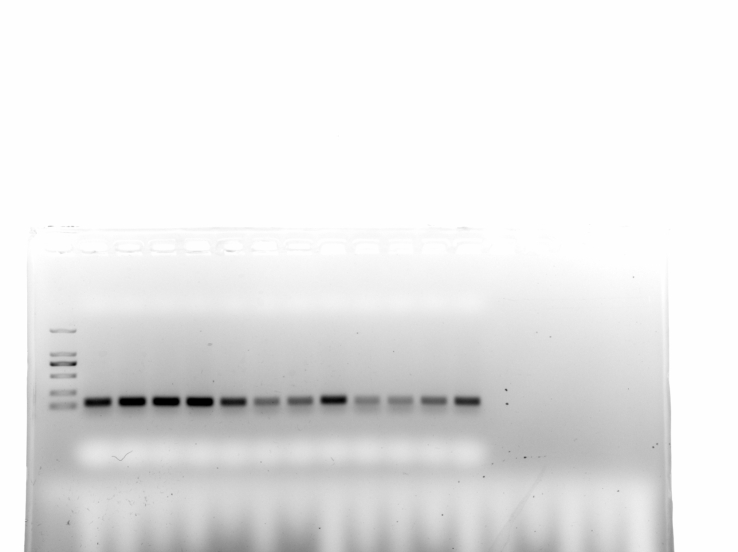


**figs6:**
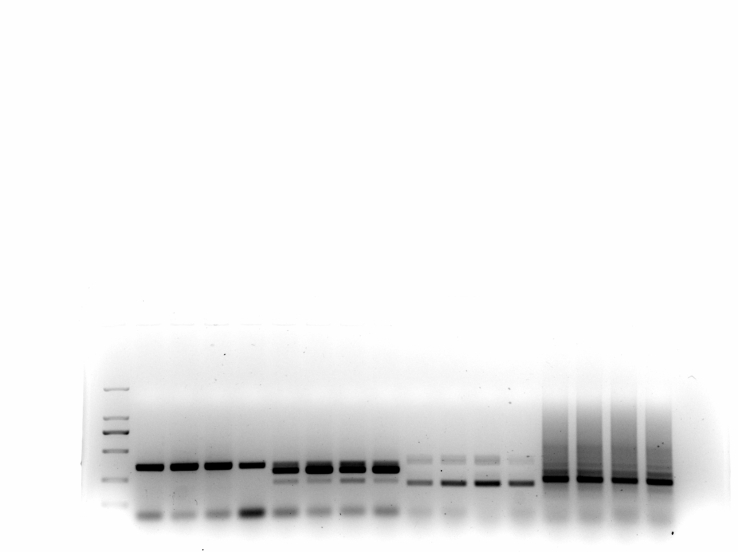


**figs7:**
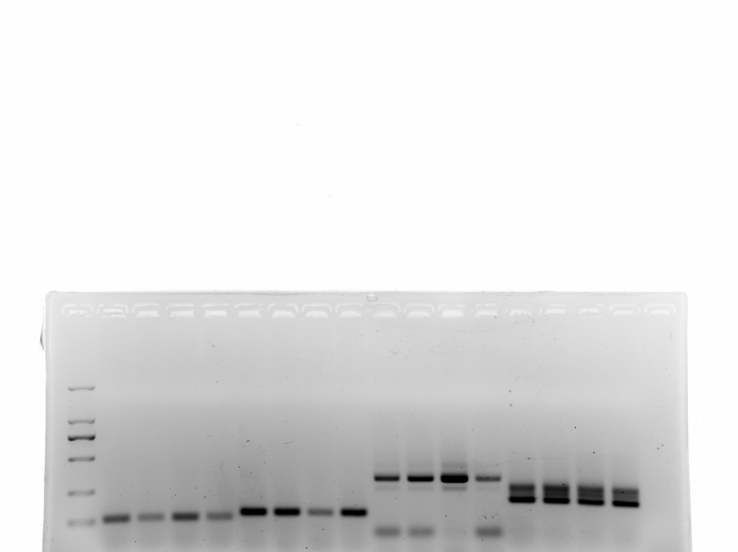


**figs8:**
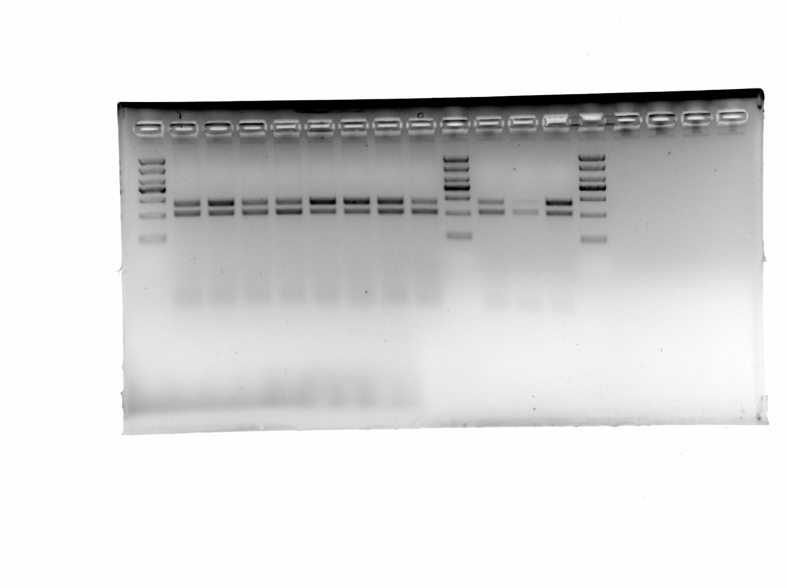


**figs9:**
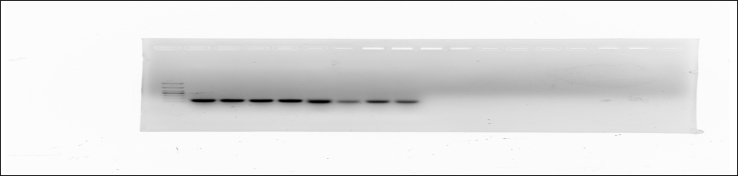


**figs10:**
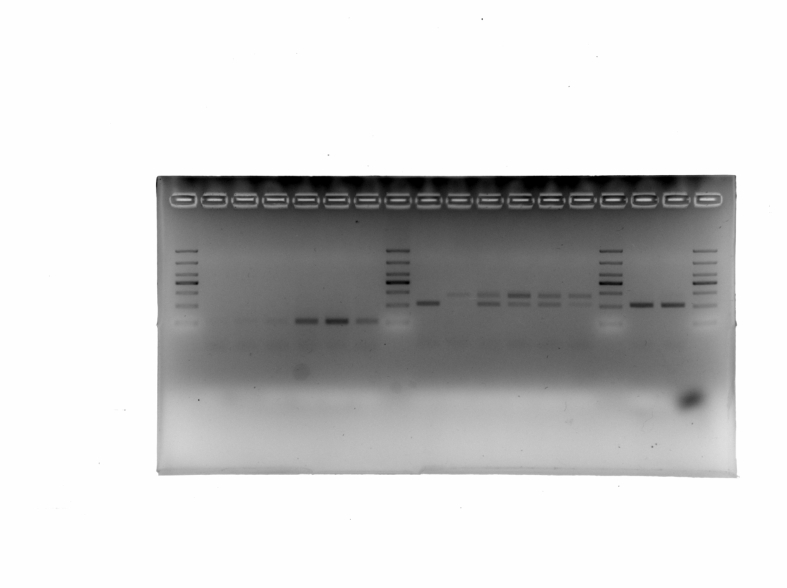


**figs11:**
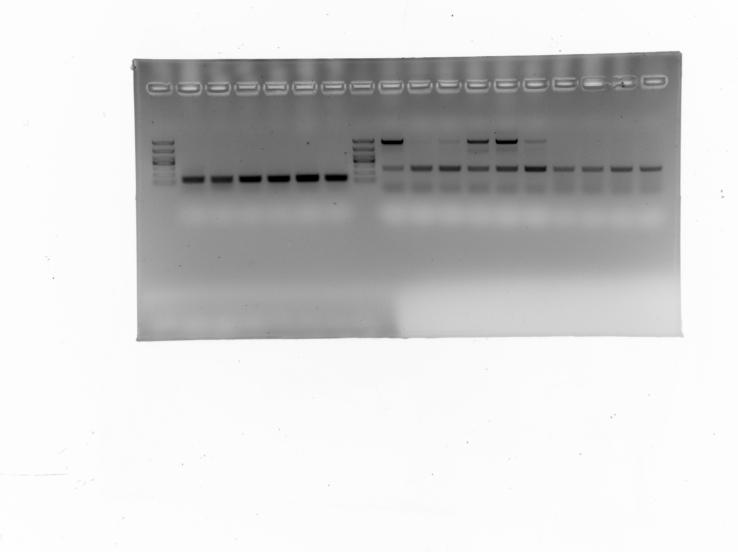


**figs12:**
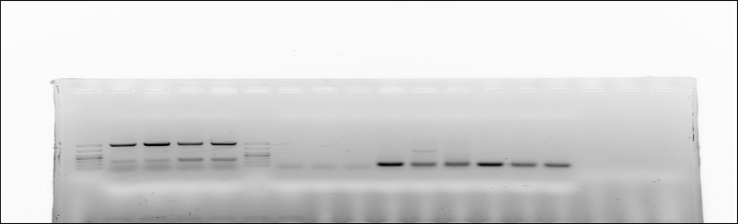


**figs13:**
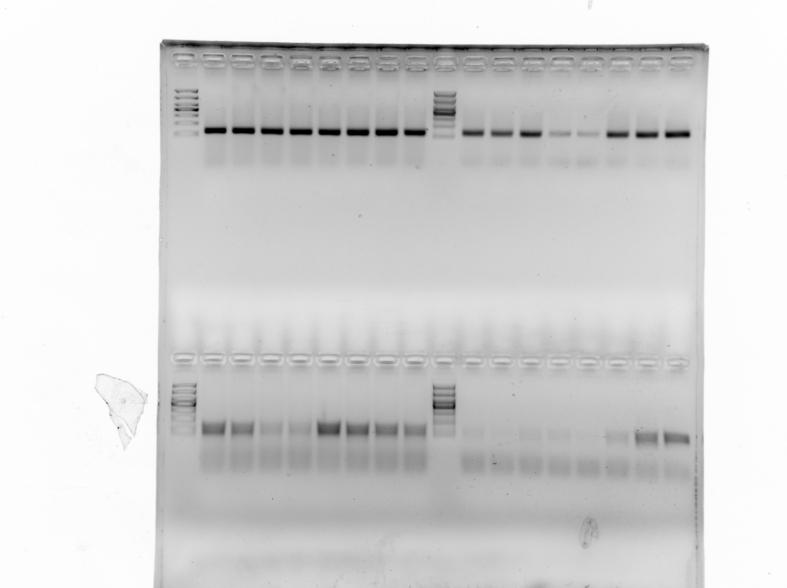


**figs14:**
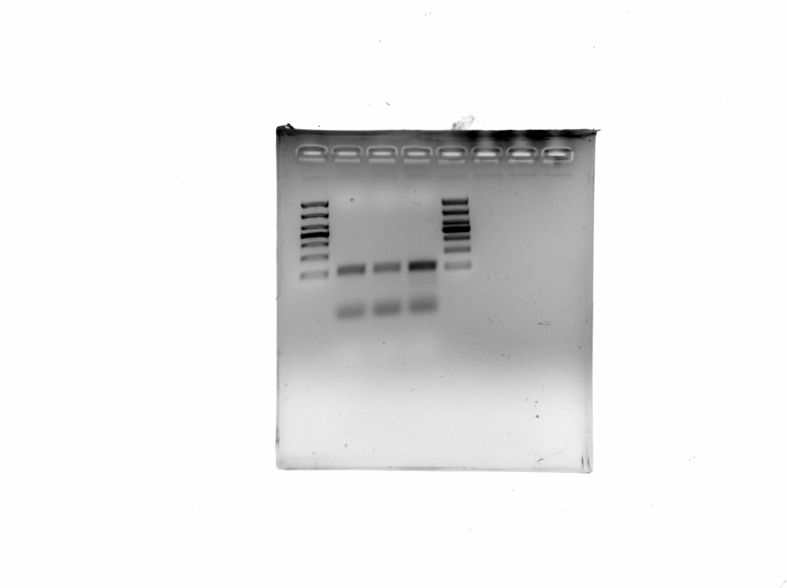


**figs15:**
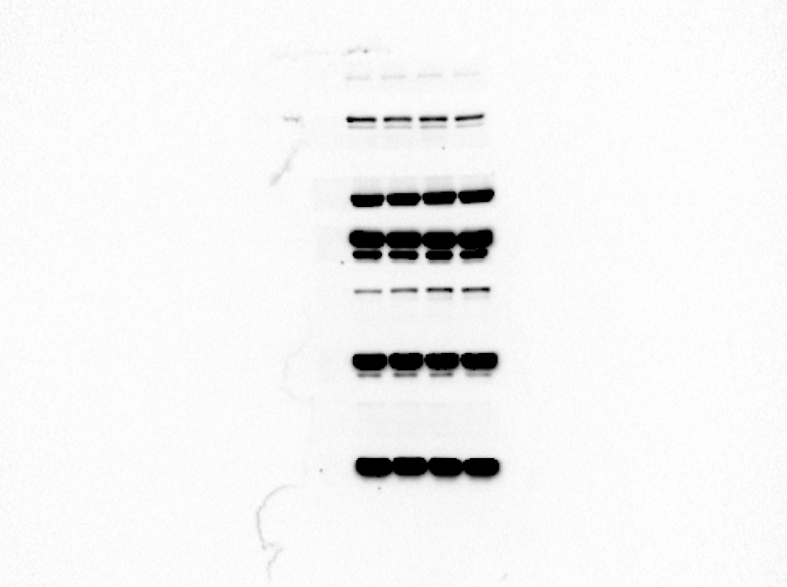


**figs16:**
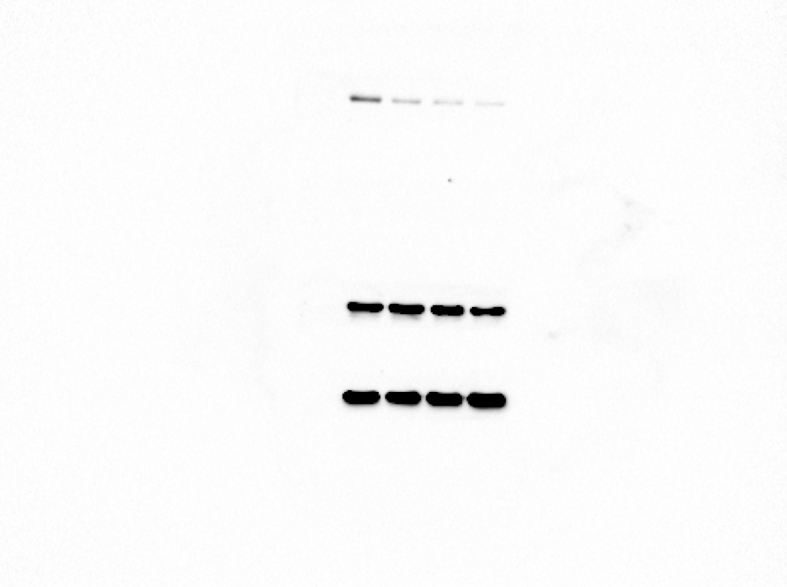


**figs17:**
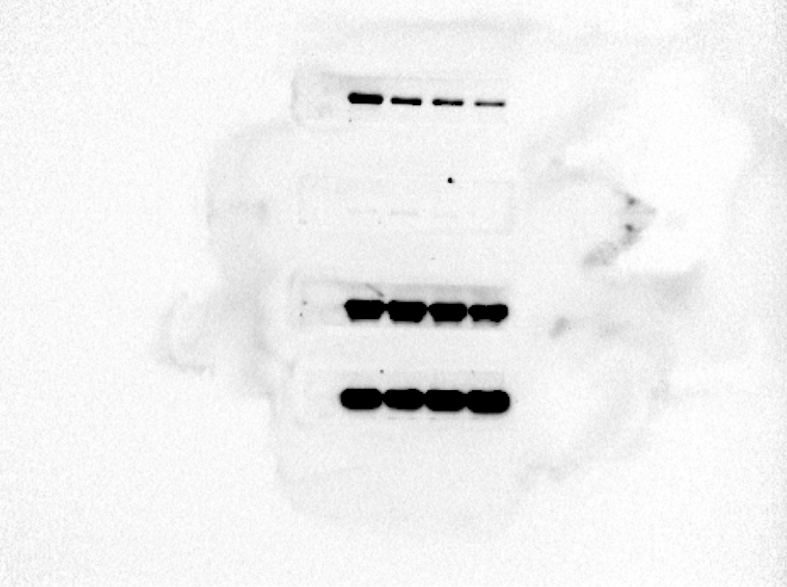


**figs18:**
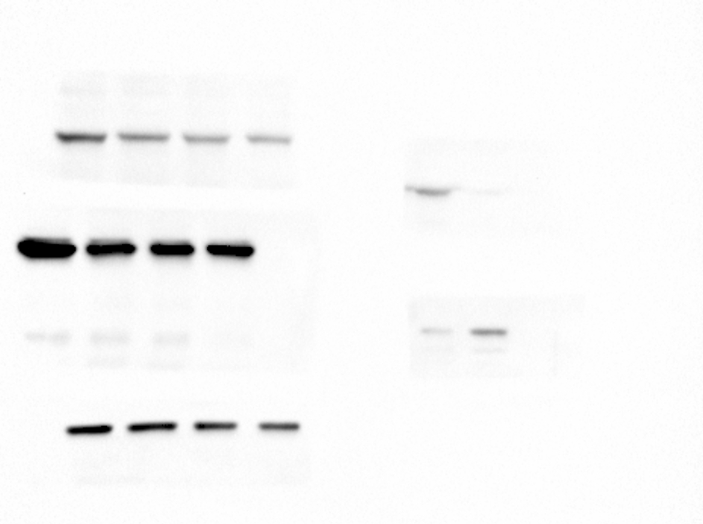


**figs19:**
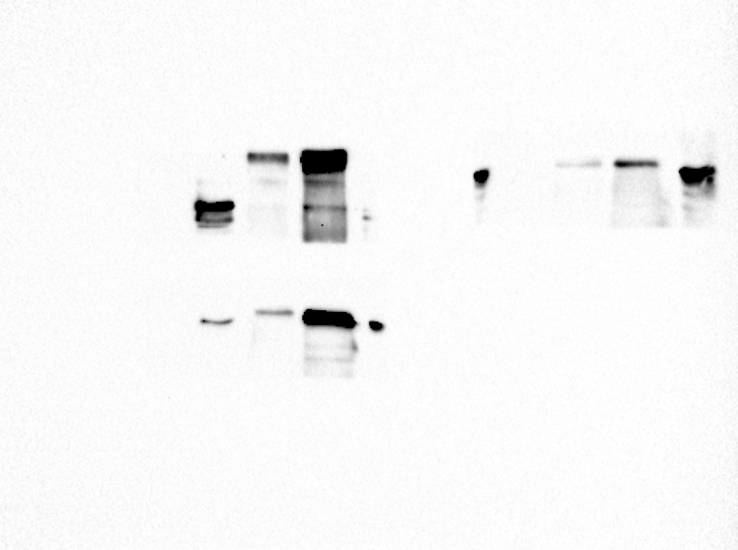


**figs20:**
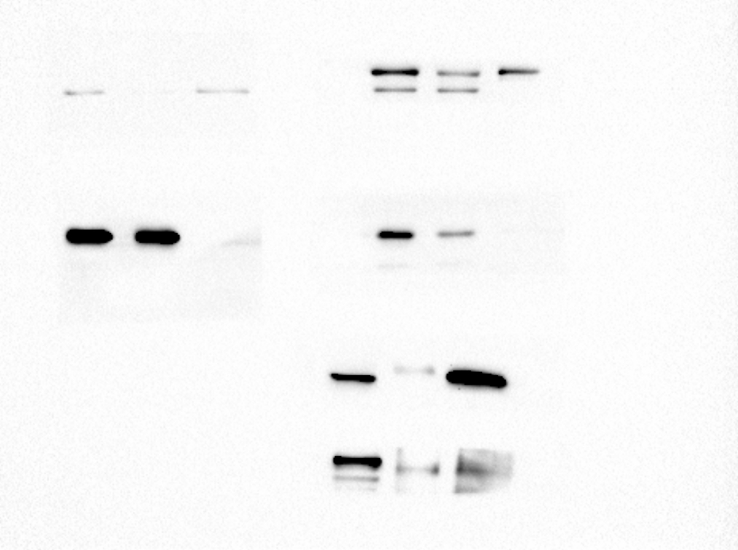


**figs21:**
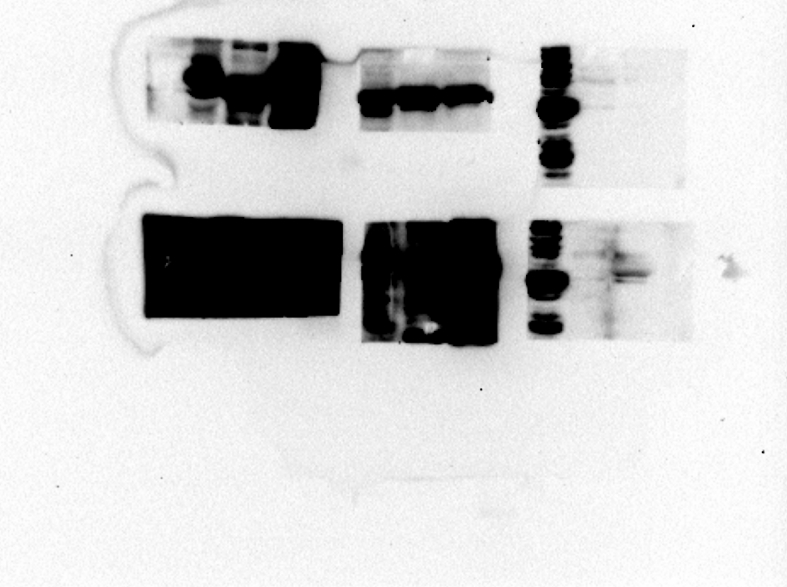


**figs22:**
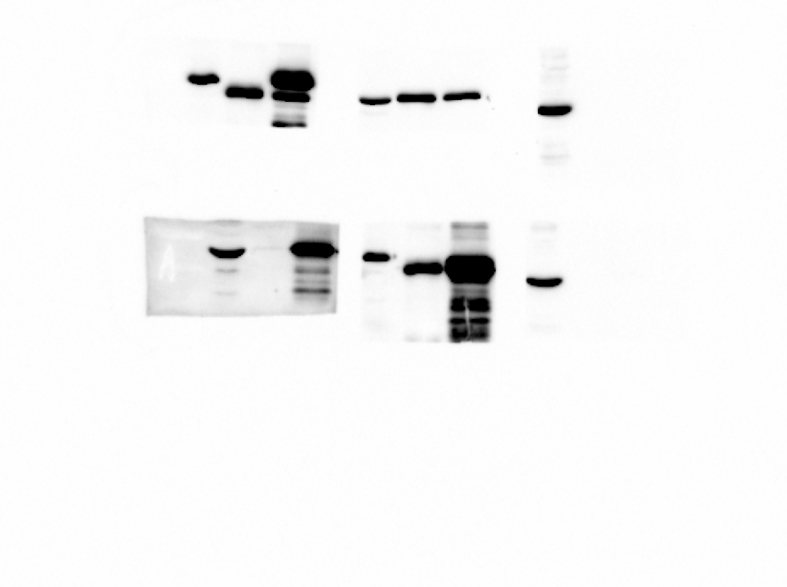


**figs23:**
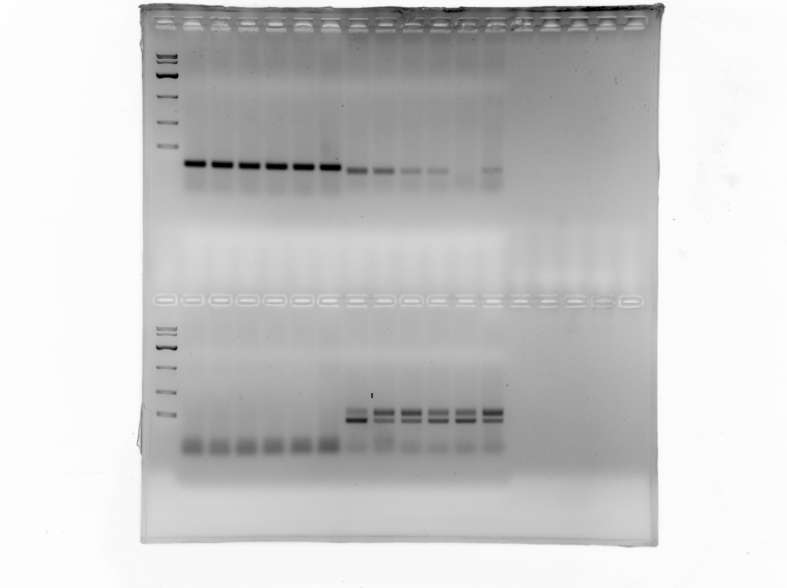


**figs24:**
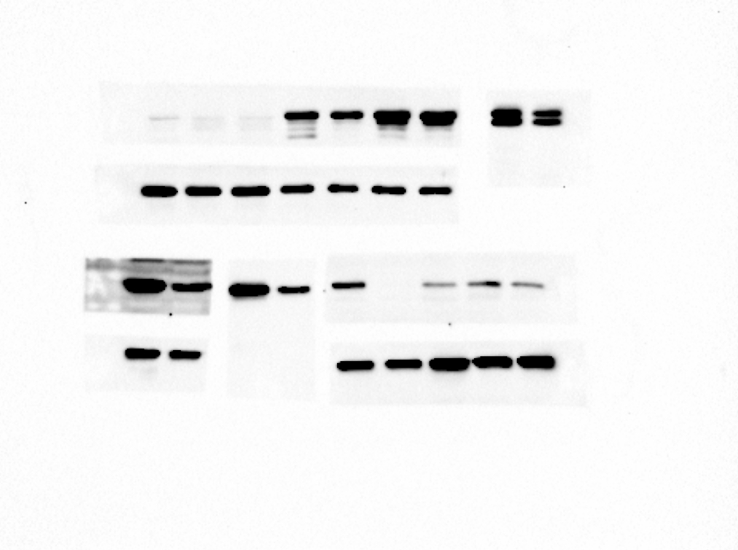


**figs25:**
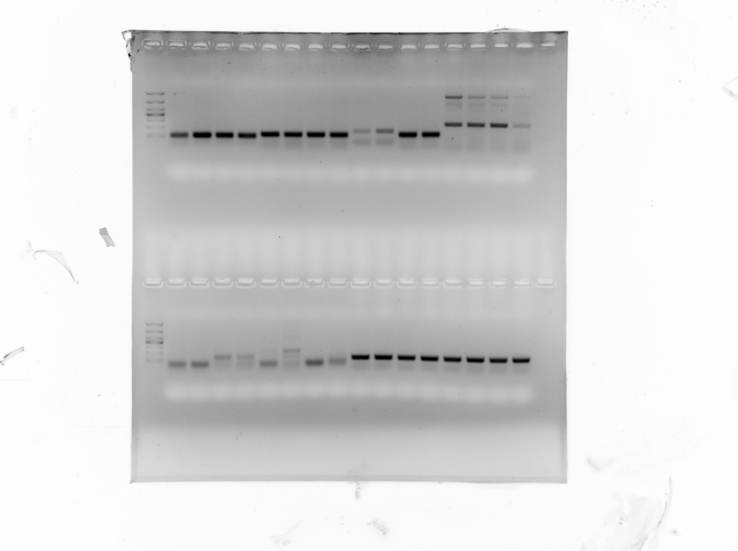


**figs26:**
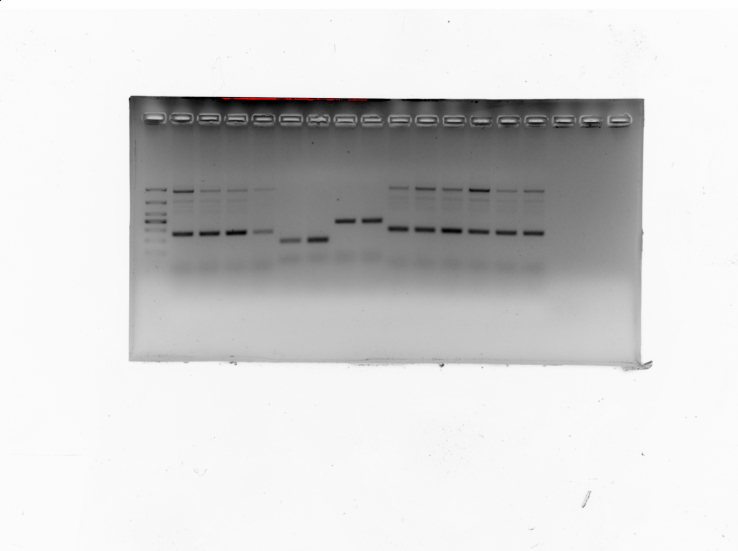


**figs27:**
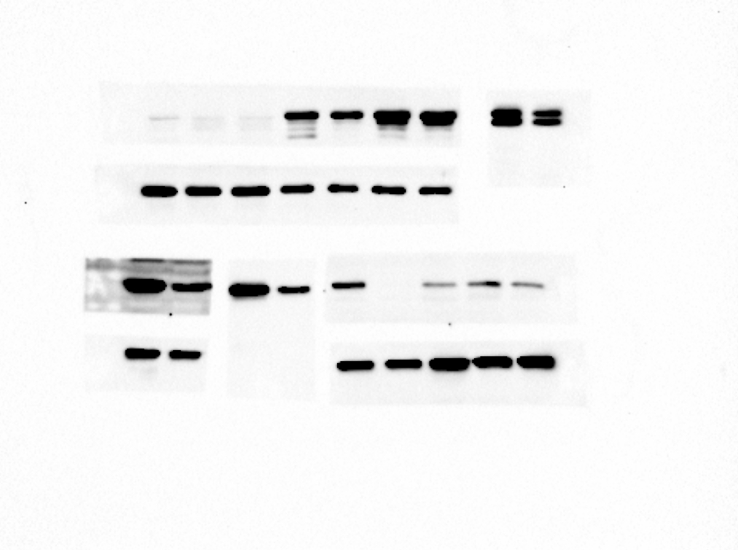


**figs28:**
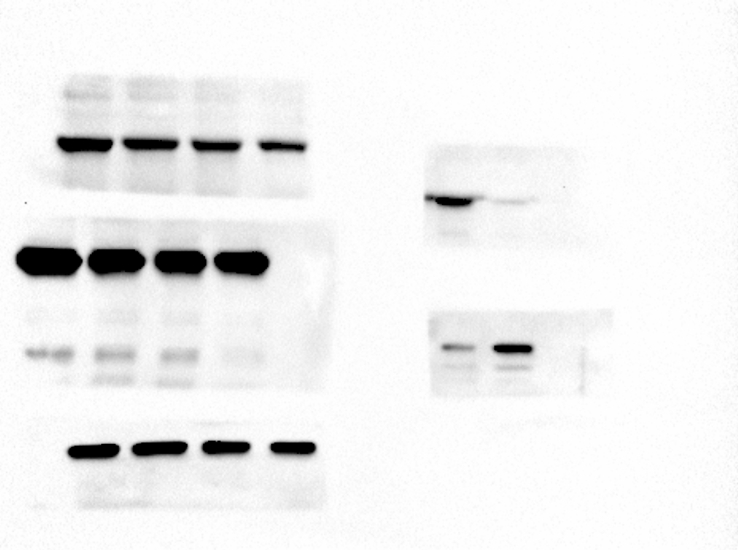


**figs29:**
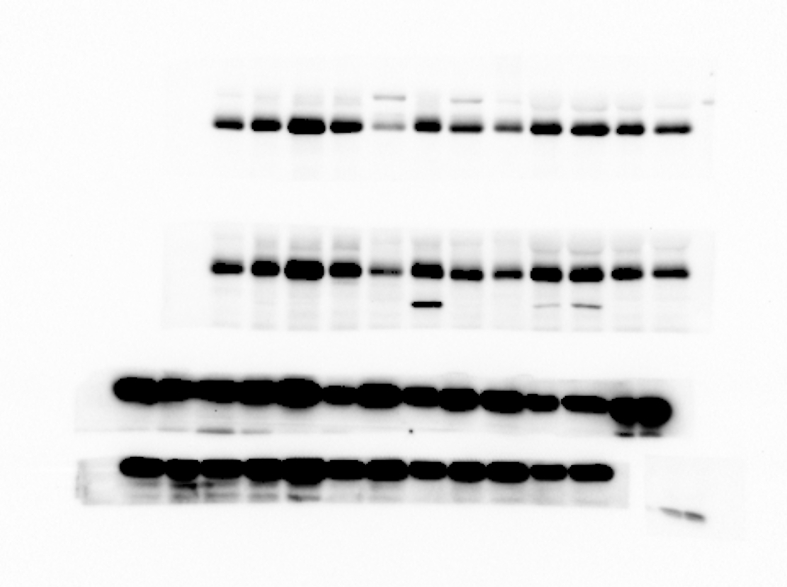


**figs30:**
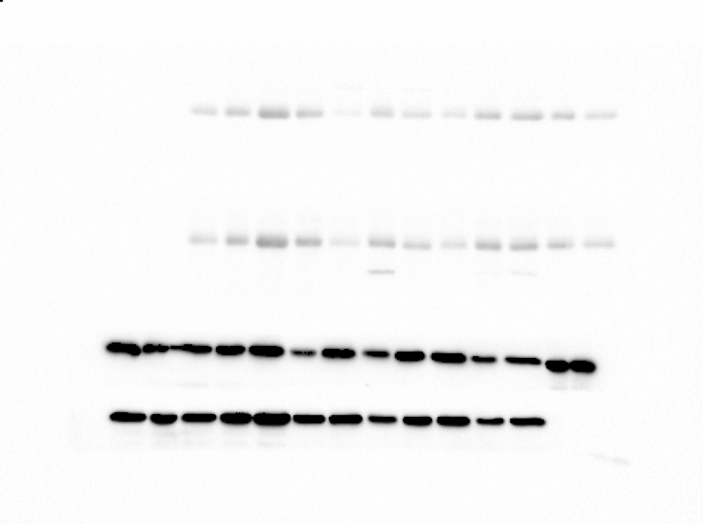


**figs31:**
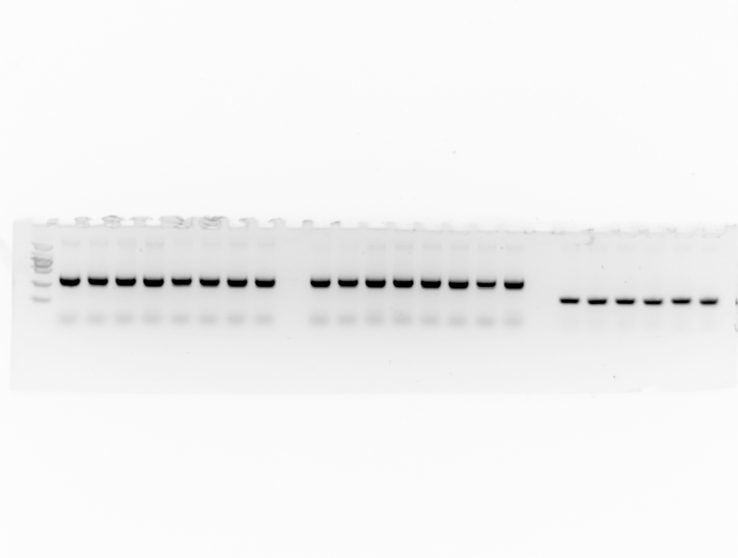


**figs32:**
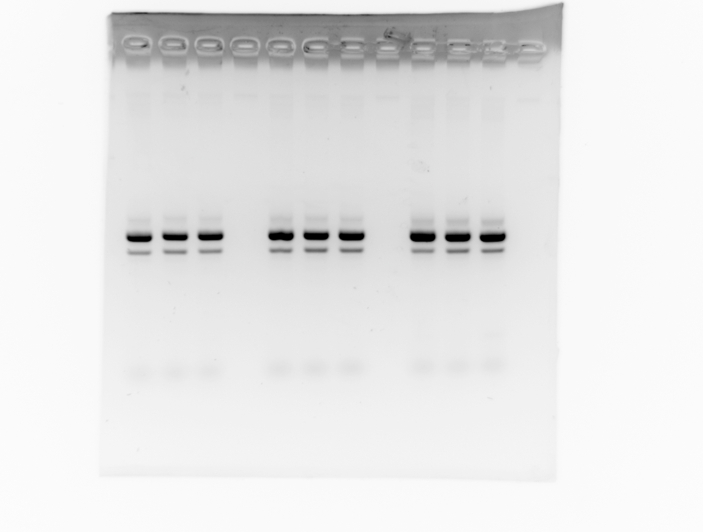


**figs33:**
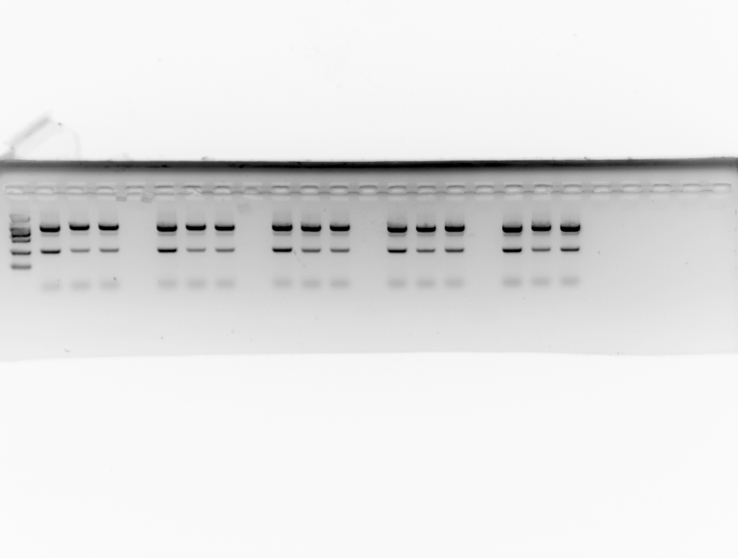


**figs34:**
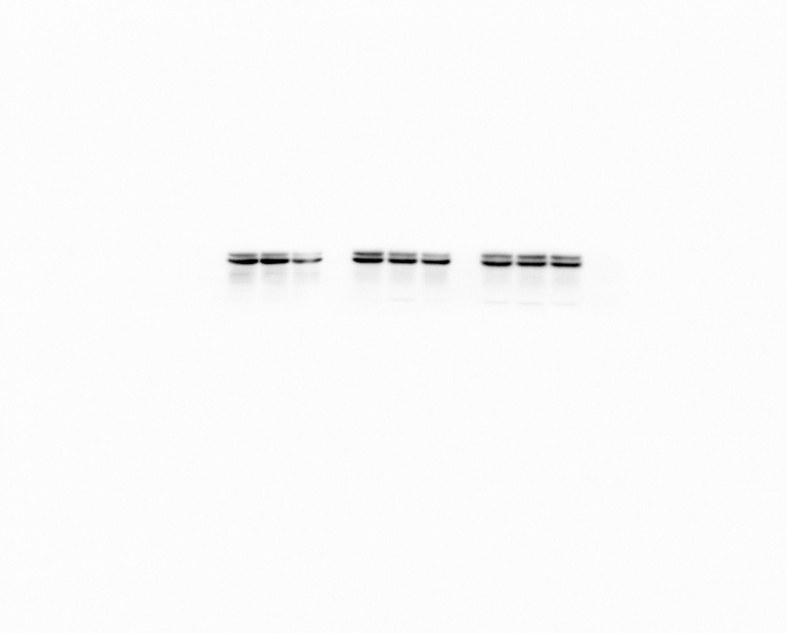


**figs35:**
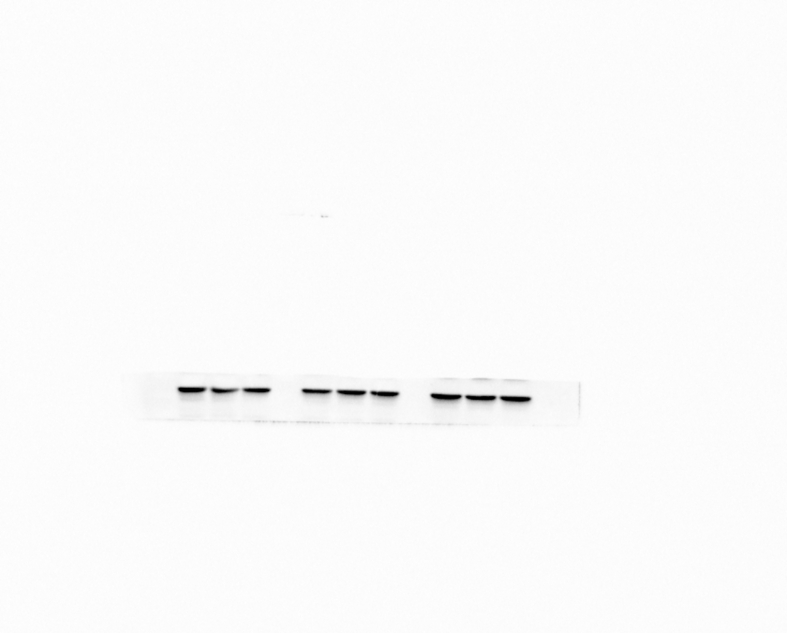


**figs36:**
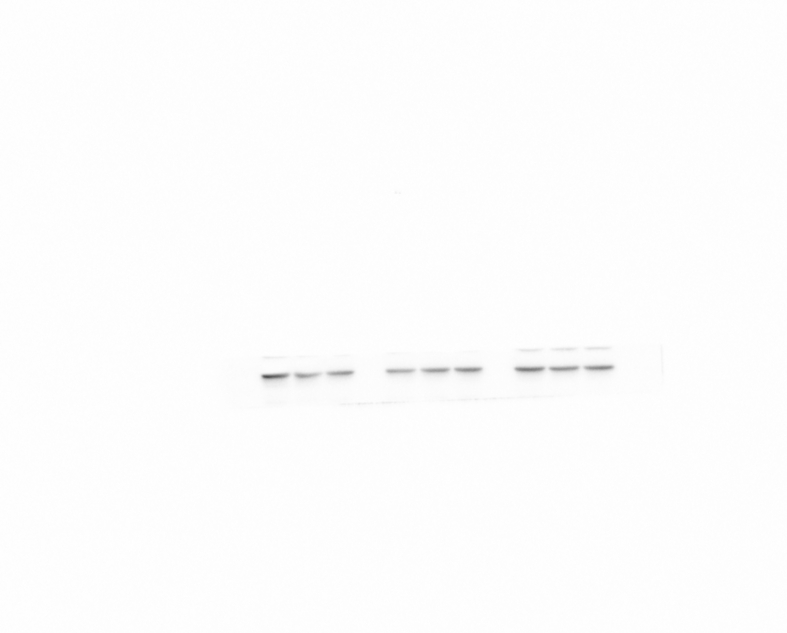


**figs37:**
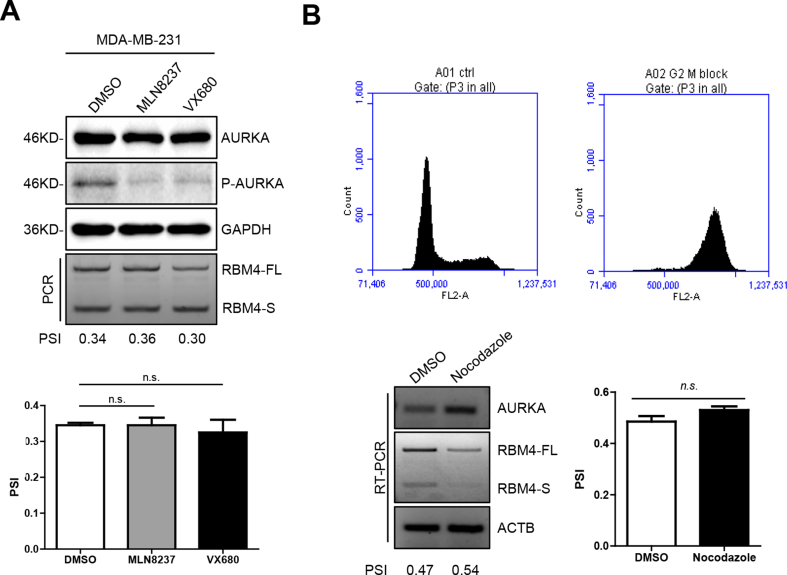


**figs38:**
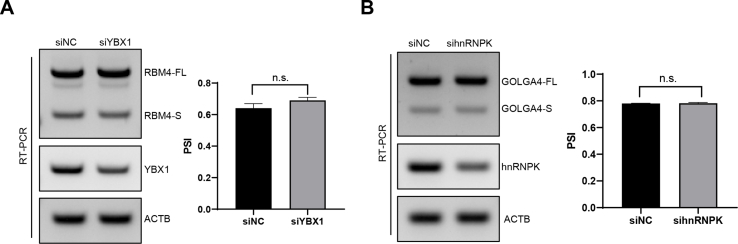


**figs39:**